# Carvedilol Prevents Ovariectomy-Induced Myocardial Contractile Dysfunction in Female Rat

**DOI:** 10.1371/journal.pone.0053226

**Published:** 2013-01-07

**Authors:** Eduardo Hertel Ribeiro, Felipe F. Potratz, Brunella M. M. Pavan, Ludimila Forechi, Filipe Lugon Moulin Lima, Jonaina Fiorim, Aurelia Araujo Fernandes, Dalton Valentim Vassallo, Ivanita Stefanon

**Affiliations:** 1 Department of Physiological Sciences, Federal University of Espirito Santo, Vitoria, Brazil; 2 Department of Physiological Sciences, EMESCAM, Vitoria, Brazil; Medical University Innsbruck, Austria

## Abstract

Carvedilol has beneficial effects on cardiac function in patients with heart failure but its effect on ovariectomy-induced myocardial contractile dysfunction remains unclear. Estrogen deficiency induces myocardial contractile dysfunction and increases cardiovascular disease risk in postmenopausal women. Our aim was to investigate whether carvedilol, a beta receptor blocker, would prevent ovariectomy-induced myocardial contractile dysfunction. Female rats (8 weeks old) that underwent bilateral ovariectomy were randomly assigned to receive daily treatment with carvedilol (OVX+CAR, 20 mg/kg), placebo (OVX) and SHAM for 58 days. Left ventricle papillary muscle was mounted for isometric tension recordings. The inotropic response to Ca^2+^ (0.62 to 3.75 mM) and isoproterenol (Iso 10^−8^ to 10^−2 ^M) were assessed. Expression of calcium handling proteins was measured by western blot analysis. Carvedilol treatment in the OVX animals: prevented weight gain and slight hypertrophy, restored the reduced positive inotropic responses to Ca^2+^ and isoproterenol, prevented the reduction in SERCA2a expression, abolished the increase in superoxide anion production, normalized the increase in p22^phox^ expression, and decreased serum angiotensin converting enzyme (ACE) activity. This study demonstrated that myocardial contractile dysfunction and SERCA2a down regulation were prevented by carvedilol treatment. Superoxide anion production and NADPH oxidase seem to be involved in this response.

## Introduction

Cardiovascular diseases are the leading cause of death in developed countries and are more prevalent in men as compared to premenopausal women [1]–[4]. Despite aggressive diagnosis and treatment, cardiovascular diseases still remain a major and growing public health problem [5].

It is well known that estrogen is linked to cardiovascular protection in premenopausal women whereas hormone replacement therapy (HRT) may have detrimental effects on cardiovascular function [6]–[8]. The use of HRT failed to reduce cardiovascular risks and controversies regarding the safety of HRT have drawn attention to new therapies for postmenopausal women [9], [10].

Estrogen deficiency plays a role in the contractile activity of the heart [11]–[13] most likely by decreasing SERCA2a expression and its function, decreasing Ca^2+^ responsiveness of myofilament activation and increasing phospholamban expression [1], [9], [14]. The decline in cardiac contractile function is characterized mainly by altered excitation-contraction coupling and impaired myofilament calcium responsiveness [1], [15]. Recently we demonstrated that myocardial contractile dysfunction induced by ovariectomy and expression of key Ca^2+^-handling proteins were prevented by losartan treatment and that AT_1_ receptor activation is involved in this response [16].

Furthermore, estrogen decreases angiotensin converting enzyme (ACE) activity and the risk of coronary artery disease in women [17]. Recent studies showed that ACE activity appears to be affected by estrogen and the down-stream signaling of the AT_1_ receptor [18]. The reduction in ACE activity was not due to a direct interaction of estrogen with the enzyme; but rather, it seems that estrogen regulates ACE mRNA synthesis at the tissue level [19].

The renin angiotensin system plays an important role in cardiovascular regulation through angiotensin II [20]. Angiotensin II increases NADPH oxidase activity and its expression, thereby increasing reactive oxygen species (ROS), leading to cardiac remodeling and contractile dysfunction [15], [20]. Cardiomyocytes are sensitive to oxidative stress and ROS are capable of influencing cellular calcium regulation at several levels, including calcium overload and oxidation of proteins involved in calcium handling such as ryanodine receptor, SERCA2a, Na^+^-Ca^2+^ exchanger and phospholamban [21], [22]. Besides, ROS also down regulate the expression of the gene responsible for SERCA2a expression, resulting in reduced rates of relaxation/contraction cycling [22], [23]. Normalized excitation-contraction coupling is essential to maintain calcium homeostasis and cardiac function [15], [20]. Ribeiro et al. [16] demonstrated that losartan restored the positive inotropic responses to Ca^2+^ and isoproterenol, prevented the reduction in SERCA2a levels and the increase in phospholamban (PLB) expression, abolished the increase in superoxide anion and normalized the increase in p22^phox^ expression in isolated papillary muscle from ovariectomized rats.

On the other hand, carvedilol, a beta receptor blocker has been widely used in humans for the treatment of heart failure once it improves symptoms and cardiac performance, with a marked reduction in cardiovascular events after onset of the disease [22]–[25].

Part of the cardioprotection afforded by carvedilol is due to its antioxidant activity, tenfold greater than any antioxidant such as vitamin E [22] and by the interaction between either the β-1 or β-2 adrenergic and AT_1_ receptor. Interfering with the signaling of one receptor, i.e. selective β adrenergic receptor blockade inhibits signaling of AT_1_ receptor, resulting in the inhibition of signaling by the reciprocal, interacting receptor [26]. Carvedilol also prevents ROS-induced decrease in SERCA2a expression [23]. Sarcoplasmatic reticulum Ca^2+^-adenosine triphosphatase (SERCA2a) and phospholamban are the major proteins responsible for intracellular calcium homeostasis [9], [27]–[30]. Carvedilol also prevents lipid and protein peroxidation induced by oxygen radicals; decreases oxidative stress and calcium overload [22]–[25], [31]. Thus, our hypothesis is that carvedilol treatment could improve contractile dysfunction induced by estrogen deficiency.

The goal of this study was to investigate whether carvedilol would prevent ovariectomy-induced myocardial contractile dysfunction. We hypothesized that carvedilol would prevent myocardial contractile dysfunction through a decrease in oxidative stress. *Therefore,* we analyzed the effects of this treatments on the following: weight gain and hypertrophy induced by ovariectomy; hemodynamic and myocardial contractility; reactive oxygen species production; measurement of plasma ACE activity and expression of key Ca^2+^-handling proteins. Our findings provide evidence that carvedilol prevents myocardial contractile dysfunction in ovariectomized female by decreasing the renin angiotensin system activity.

## Materials and Methods

### Animal Care

The care and use of the laboratory animals were in accordance with National Institutes of Health (NIH) guidelines and were approved by the Institutional Ethics Committee of the Health Science Center of Vitória- EMESCAM. All rats had free access to tap water and food.

### Experimental Groups, Surgical Procedures and Treatment

Eight-week-old female Wistar rats were randomly divided into three groups. Rats underwent bilateral ovariectomy as described previously [9], [32]. Briefly, rats were anesthetized, and a dorsal midline skin incision was made caudal to the posterior border of the ribs. The posterior abdominal muscle wall was bluntly dissected, the abdominal cavity was opened and the ovary was gently exteriorized and removed. The uterine horn was returned to the abdomen. The skin incision was closed with sterile nylon sutures, and the process was repeated on the other side. The rats were randomly assigned to receive treatment with carvedilol (OVX+CAR, 20 mg/kg in a 0.05% sodium carboxymethylcellulose solution and administered through canula by an oral route, daily) or placebo (OVX), and the last group underwent a SHAM operation and served as normal controls. All treatments lasted for 58 days.

Left ventricle papillary muscle contractility was studied 60 days postsurgery. At the time of sacrifice, the adequacy of the ovariectomy was grossly determined by the absence of ovarian tissue and marked uterine atrophy. We also determined the weight of the entire animal, as well as the weight of the left ventricle and the uterus.

### Isometric Tension and Myocardial Contractility

Rats received 500 units of heparin intraperitoneally (i.p.) and were anesthetized 10 minutes later with urethane 1.2 g/kg (Sigma). The hearts were rapidly removed and perfused through the aortic stump as the left ventricle papillary muscles were dissected. Muscle preparations were mounted for isometric tension recording and maintained in 20 mL Krebs–Henseleit solution (in mM: NaCl 118, KCl 4.7, CaCl_2_ 1.25, KH_2_PO_4_ 1.2, MgSO_4_ 1.2, NaHCO_3_ 23 and glucose 11) at 30°C and pH  = 7.4, which was continuously aerated with 95% O2 and 5% CO_2_. Resting tension was adjusted to produce maximal contractile force (Lmax). The twitch contraction rate was controlled by isolated rectangular pulses (10 to 15 V, 12 ms duration) through a pair of platinum electrodes. The standard stimulation rate was 0.5 Hz (steady-state). Isometric force development was measured with an isometric force transducer (TSD105A, Biopac) and normalized to muscle weight (g/g). Recording started after 30 minutes to permit the muscle to adapt to the new environmental conditions. Myocardial contractility was tested by measuring the positive inotropic response to changes in extracellular calcium (CaCl_2_, Merck) concentration (0.62 to 3.75 mM) and by adding isoproterenol (Sigma) concentrations to the bath (10^−8^–10^−2^ M).

### Hemodynamic and Ventricular Compliance Measurements

Animals were anesthetized with a mixture of the ketamine/xylazine (90 mg/kg and 10 mg/kg, respectively). The right carotid artery was isolated, and a polyethylene catheter (PE-50) was inserted and connected to a data acquisition system to measure the hemodynamic parameters. Following an adaptation period of 30 min, arterial pressure acquisition started. The catheter was advanced into the left ventricle, and systolic pressures (LVSP), end-diastolic pressures (LVEDP), dP/dt +, dP/dt – and heart rate (HR) were continuously monitored for 15 min. To define the passive pressure-volume characteristics of the left ventricle, the heart was arrested in diastole with potassium chloride (1 M) and a double lumen catheter (PE 50 inside PE 200) was inserted 6 mm into the left ventricle via the aorta. The right ventricular free wall was incised to avoid fluid accumulation and variable compressive force on the interventricular septum. The atrioventricular groove was tied, and the ventricle was manually compressed to expel blood and create a negative pressure of −5 mm Hg, which was taken as zero volume. Physiological saline was infused at 0.68 mL/min via one lumen, while the intraventricular pressure was continually recorded through the other lumen over the pressure range of −5 to 30 mmHg. At least three reproducible pressure-volume curves were obtained within 10 minutes of cardiac arrest and well before the onset of rigor mortis. Ventricular volumes at pressures of 0, 2.5, 5, 10, 15, 20, and 30 mmHg were determined from the pressure-volume curve.

The obtained curves were segmented and then separately analyzed. Pressure follows a linear pattern from 0 to 5 mmHg during volume infusion, and the slope is indicative of left ventricular dilatation. For the 5 to 30 mmHg segment, the curve was adjusted to a monoexponential model. Thus, to determine the stiffness constant during the 5–30 mmHg intervals, a logarithmic transformation was performed on the pressure scale to create a linear fit.

### Western Blot Analysis

Western blot was performed as previously described [29], [33], [34]. Proteins from homogenized hearts (50 µg for PLB and 80 µg for the other proteins) were separated by 7.5%, 10% or 15% SDS-PAGE. Proteins were transferred onto nitrocellulose membranes, which were incubated with mouse monoclonal antibodies for SERCA2a (1∶1000, Affinity BioReagents, CO, USA), PLB (0.5 µg/ml, Affinity BioReagents, CO, USA), phosphorylated PLB at serine 16 (1∶5000, Badrilla, UK), phosphorylated PLB at threonine 17 (1∶5000, Badrilla, UK), AT_1_ receptor (1∶1000, Santa Cruz Biotechnology) and AT_2_ receptor (1∶750, Santa Cruz Biotechnology). After washing, the membranes were incubated with anti-mouse (1∶5000, Stressgen, Victoria, Canada) or anti-rabbit (1∶7000, Stressgen, Victoria, Canada) immunoglobulin antibodies conjugated to horseradish peroxidase. After thorough washing, immunocomplexes were detected using an enhanced horseradish peroxidase/luminal chemiluminescence system (ECL Plus, Amersham International, Little Chalfont, UK) and film (Hyperfilm ECL International). Signals on the immunoblot were quantified with the NIH Image V1.56 computer program. Each membrane was reprobed to determine GAPDH expression using a mouse monoclonal antibody (1∶5000, Abcam Cambridge, MA, USA).

### *In situ* Detection of Papillary O_2_^–^ Production

The oxidative fluorescent dye dihydroethidium (DHE) was used to evaluate O_2_^–^ production in situ, as previously described [35]. Dihydroethidium freely permeates cells and is oxidized in the presence of O_2_^–^ to ethidium bromide, which is trapped by intercalation with DNA. Ethidium bromide is excited at 546 nm and emits at 610 nm. Frozen tissue segments were cut into 10-µm-thick sections and placed on glass slides. Serial sections were equilibrated under identical conditions for 30 min at 37°C in Krebs-HEPES buffer (in mM: 130 NaCl, 5.6 KCl, 2 CaCl_2_, 0.24 MgCl_2_, 8.3 HEPES, and 11 glucose, pH  = 7.4). Fresh buffer containing DHE (2 µM) was applied topically to each tissue section, covered with a cover slip, incubated for 30 min in a light-protected humidified chamber at 37°C, and then viewed with a fluorescent microscope (Nikon, 200× magnification) using the same imaging settings for all sections. Fluorescence was detected with a 568-nm long-pass filter. For quantification, 8 tissue sections per animal were sampled and averaged for each experimental condition. The mean fluorescence densities in the target region were calculated.

### Measurement of MDA Production

Plasmatic malondialdehyde (MDA) levels were measured with a modified thiobarbituric acid (TBA) assay [35]. Plasma or powdered heart tissue was mixed with 20% trichloroacetic acid in 0.6 M HCl (1∶1 vol/vol), and tubes were incubated on ice for 20 min to precipitate plasma components that present possible interferences. Samples were centrifuged at 1,500×*g* for 15 min before adding TBA (120 mM in 260 mM Tris, pH = 7) to the supernatant in a proportion of 1∶5 (vol/vol), and the mixture was boiled at 97°C for 30 min. Spectrophotometric measurements at 535 nm were performed at 20°C.

### Measurement of Plasma ACE Activity

Angiotensin converting enzyme activity was measured with a fluorometric method [34]. Briefly, triplicate plasma samples (3 µL) or 75 ug of cardiac homogenized tissue were incubated for 15 minutes at 37°C with 40 µL of assay buffer containing the ACE substrate 5 mM Hip-His-Leu (Sigma). The reaction was stopped by adding 190 µL of 0.35 N HCl. The resulting product (His-Leu) was fluorometrically measured following 10 min incubation with 17 µL of 2% o-phatal-dialdehyde in methanol. Fluorescence measurements were carried out at 37°C in a plate reader (Synergy 2, Biotek) with 350 nm excitation and 520 nm emission filters. The fluorescence plate reader was controlled by the Gen 5 Software. Black 96-well polystyrene microplates (Corning Incorporated, NY, USA) were used. A calibration curve with His-Leu (Sigma) was included in each plate.

### Immunohistochemical Analysis

Immunohistochemical localization of p22^phox^ was determined in 10-µm sections of frozen papillary muscle using an anti-p22^phox^ (1∶50, Santa Cruz Biotechnology) antibody. Briefly, frozen sections were fixed in cold acetone for 5 minutes, followed by pretreatment with 0.3% hydrogen peroxide for 20 minutes to inhibit endogenous peroxidase activity. Subsequently, sections were blocked with 5% horse serum for 60 min and incubated with the primary antibody overnight at 4°C. After rinsing with PBS, the sections were incubated for 30 min with a biotinylated secondary antibody, followed by incubation with avidin-biotinylated horseradish peroxidase complex (Vectastain ABC, Vector). Serial sections treated with nonimmune IgG did not show any staining. Peroxidase staining was detected using the DAB detection system (Vector Laboratories).

### Cardiac Interstitial Collagen Quantification

Tissue samples from left and right ventricles were dehydrated, embedded in paraffin and cut in sections of 5 µm thickness. These sections were stained with picrosirius red staining (Aldrich Chemical Company). Interstitial collagen quantifications in the left ventricles were performed using an image analysis system (BEL Engineering, Top Light B2, Italy). The area of interstitial fibrosis was identified after excluding the vessel area and scar area from the region of interest and determined as the ratio of interstitial collagen deposit to the total remaining tissue area from each ventricle. For each sample, 10 to 15 fields were analyzed with a 40× objective lens under transmitted light. All procedures were performed in samples obtained from middle transversal segments of the heart.

### Statistical Analysis

All values are expressed as the means ± SEM. Differences among groups were analyzed using one- or two-way ANOVA, repeated measures followed by the Tukey post hoc test for multiple comparisons. A p-value <0.05 was considered significant. For protein expression, the data are expressed as the ratio between the protein of interest and GAPDH.

### Drugs and Chemicals

All chemicals, unless otherwise specified, were purchased from Sigma Chemical (St. Louis, MO) or Merck (Germany).

## Results

### Body, Heart and Uterus Weight

The body weights of ovariectomized rats were significantly greater than those of SHAM rats. Carvedilol treatment reduced weight gain after ovariectomy (Table 1). Ovariectomy and carvedilol treatment decreased left ventricle weight to body weight ratio explained by the weight gain in both groups. The left ventricle weight was found higher in the OVX group when compared to SHAM and carvedilol treatment prevented this increase (SHAM: 0.56±0.01, OVX: 0.63±0.02* and OVX+CAR: 0.54±0.01# (g); * significantly different from SHAM and # from OVX). When we evaluated the left ventricle weight to tibia length ratio, rats in the OVX group exhibited a significantly higher ratio compared to SHAM, and carvedilol treatment restored this ratio to the same level as the SHAM group (Table 1). The deficiency of estrogen in OVX and OVX+CAR rats induced a significant decrease in uterine weight compared to SHAM controls. The tibia length and papillary muscle weight did not differ among groups (Table 1).

**Table 1:** Ponderal data from SHAM, Ovariectomized and Ovariectomized plus Carvedilol: BW, body weight (n = 7–9); LV/BW, left ventricle body weight ratio (n = 9–10); Uterus/BW, uterus body weight ratio (n = 8–10); Tibia length (n = 7–10); LV/Tibia, left ventricle tibia ratio (n = 14–19) and papillary muscle weight (n = 7–9).

	SHAM	OVX	OVX+CAR
BW (g)	255±5	317±7*	294±3.6*#
LV (g)	0.52±0.01	0.63±0.02*	0.54±0.01#
LV/BW (mg/g)	2±0.02	1.94±0.05*	1.84±0.032*
Uterus/BW	2.51±0.25	0.9±0.06*	0.62±0.03*
Tibia (mm)	38.0±0.43	38.6±0.35	38±0.18
LV/Tibia (mg/mm)	14±0.22	15.5±0.4*	14.6±0.3#
Papillary Muscle (mg)	5±0.35	5.92±0.5	6.96±1.3

Data are shown as mean ± SEM.

* p<0.05 versus Sham and #p<0.05 versus OVX.

ANOVA one way and Tukey test.

### Isometric Contractile Response to Calcium

**Figure 1:**
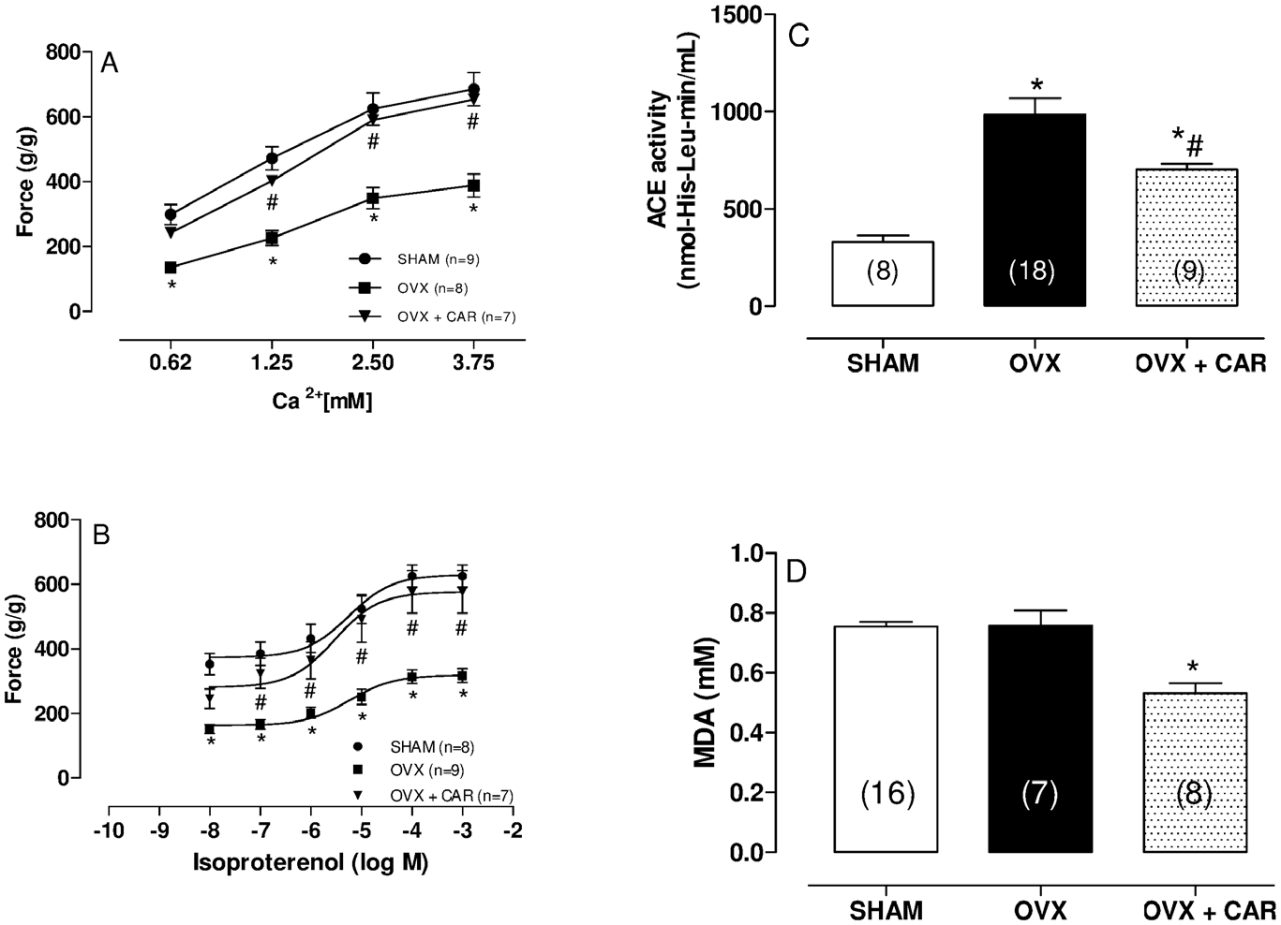
Effects of increasing extracellular calcium concentration from 0.62 to 3.75 mM and of treatment with increasing concentrations (10^−8^ to 10^−2^ M) of the β-adrenergic agonist isoproterenol on force development in isolated left ventricle papillary muscles. Angiotensin converting enzyme (ACE) activity (nmol His-Leu/ml^−1^) in serum and plasmatic malondialdehyde (MDA) levels from SHAM, ovariectomized (OVX) and ovariectomized plus carvedilol (OVX+CAR) rats 60 days postsurgery. Force (g/g) values are expressed as the means ± S.E.M. * Significantly different from SHAM and # from OVX (*p*<0.05) using two-way ANOVA, repeated measures and Tukey post hoc test.

As expected, increases in extracellular calcium concentration resulted in a positive inotropic response (Figure 1). We found a difference in the inotropic response to calcium between the OVX and SHAM groups for all concentrations studied (Figure 1A); the OVX group response is approximately 50% less compared to the SHAM rats. Interestingly, carvedilol treatment restored the reduction in the inotropic response to extracellular Ca^2+^ (Figure 1A).

### Isometric Contractile Response to β-adrenergic Receptor Stimulation

Dose-response curves were measured for isoproterenol, a nonspecific β-adrenergic agonist (Figure 1B). β-adrenergic receptor stimulation in the heart increases contractility and accelerates relaxation by activating the adenylyl cyclase/cAMP/protein kinase pathway. As expected, isoproterenol promoted a positive inotropic effect in all groups studied. However, this response was reduced in the OVX group compared to SHAM controls by approximately 50% (Figure 1B). Carvedilol treatment restored the inotropic response to isoproterenol (Figure 1B).

### Plasmatic ACE Activity, Papillary Superoxide Anion Production and Plasmatic MDA Levels

After 8 weeks, plasma ACE activity was 2.8-fold greater in the OVX compared to SHAM group. Carvedilol treatment decreased ACE activity as compared to OVX group (Figure 1C). Cardiac ACE activity was not different in the left ventricle (SHAM: 39±3.2; OVX: 37±1.3; OVX+CAR: 35±2.2 nmol-His-Leu/mL/min).

**Figure 2:**
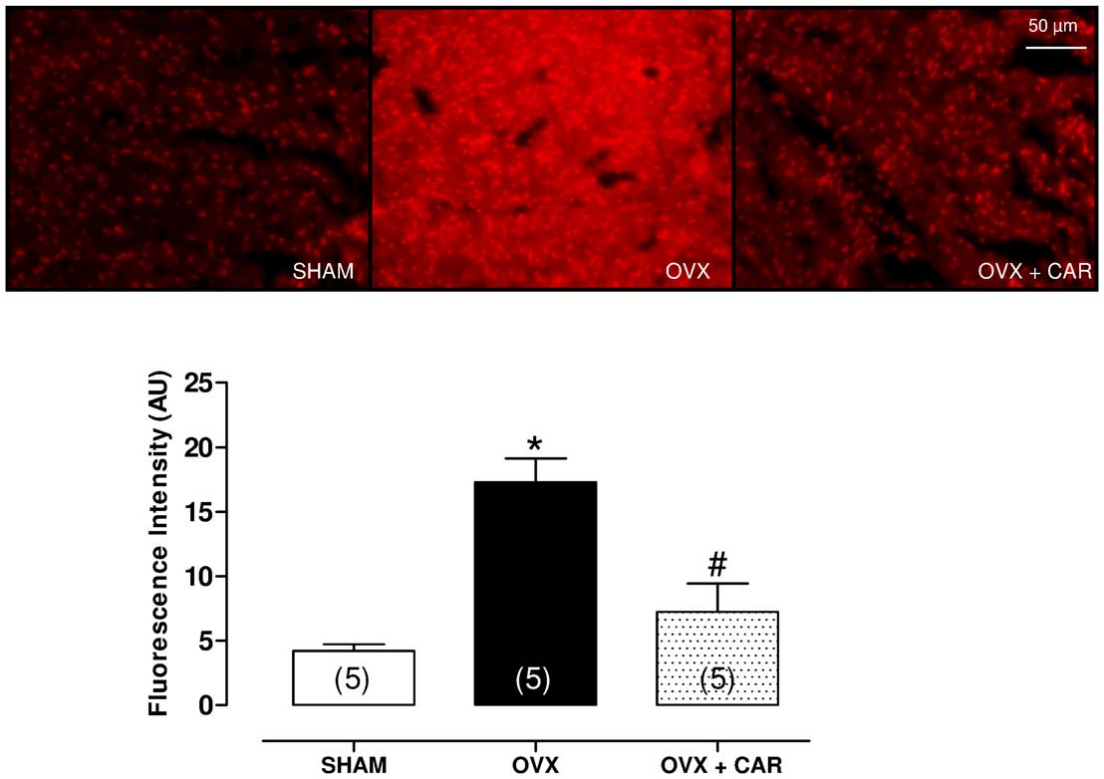
Superoxide anion production in papillary muscle of SHAM, Ovariectomized (OVX), Ovariectomized plus carvedilol (OVX+CAR) at 60 days postsurgery. The values are expressed as the means ± S.E.M. * Significantly different from SHAM and # from OVX (*p*<0.05) using one-way ANOVA and Tukey post hoc test.

Basal superoxide anion production was 4-fold higher in papillary segments from OVX rats than in SHAM rats. Carvedilol treatment prevented the increase in superoxide production in papillary segments from OVX+CAR rats (Figure 2).

Plasmatic MDA levels did not differ between SHAM and OVX groups. However, the carvedilol group exhibited a lower MDA level compared to the SHAM group (Figure 1D). Cardiac MDA levels did not differ among groups (SHAM: 0.08±0.04; OVX: 0.13±0.05; OVX+CAR: 0.08±0.02).

### Western Blot Analysis

**Figure 3:**
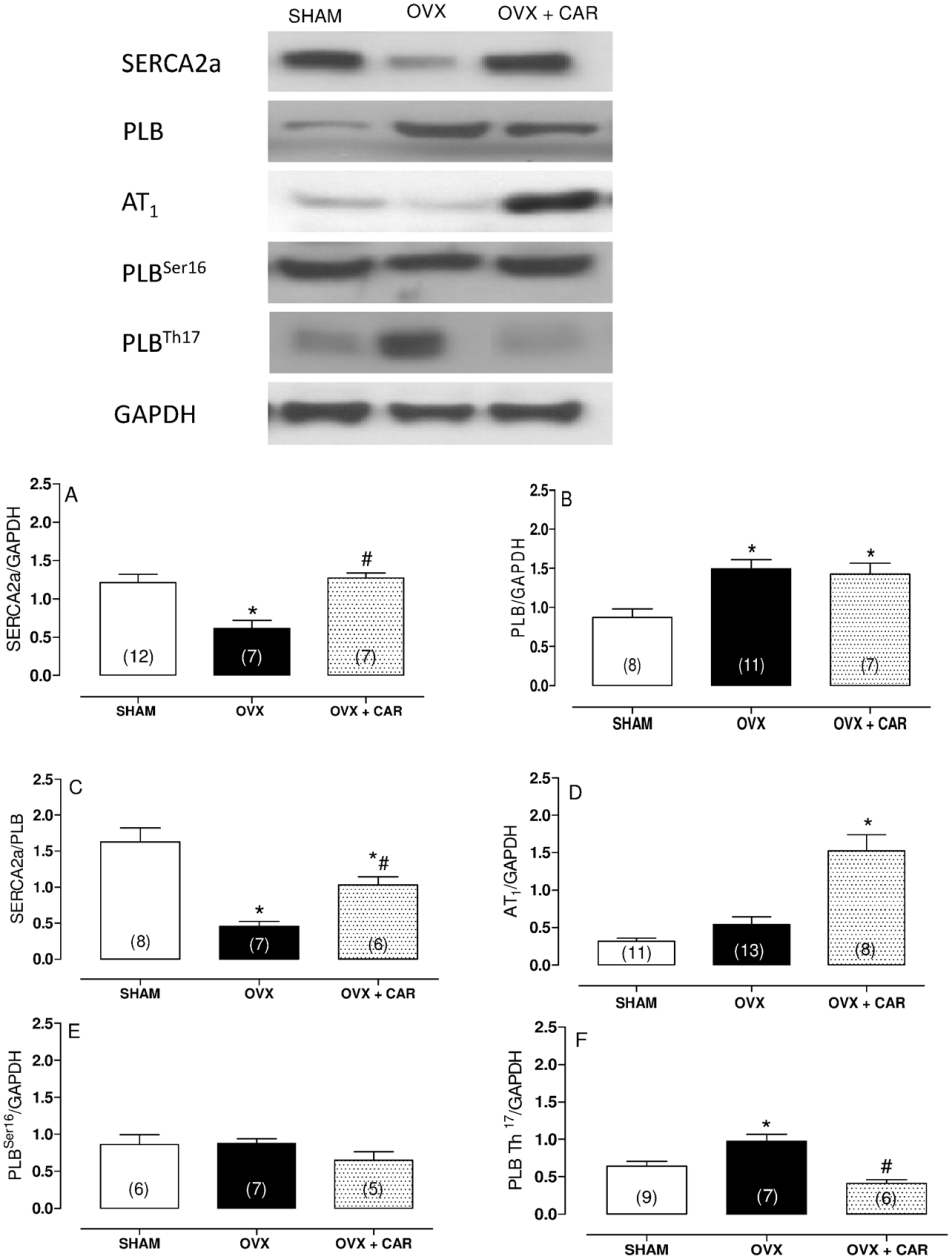
Densitometric analysis of Western blots. SR Ca^2+^-ATPase (A), (B) phospholamban (PLB), (C) SERCA2a/PLB ratio, (D) AT_1_ receptor, (E) phosphorylated phospholamban at Ser^16^ (PLBSer^16^) and phosphorylated phospholamban at Thr^17^ (PLBThr^17^) in hearts from SHAM, Ovariectomized (OVX), Ovariectomized plus carvedilol (OVX+CAR) rats. The values are expressed as the means ± S.E.M * *p*<0.05 vs. SHAM rats. # *p*<0.05 vs. OVX rats using one-way ANOVA and Tukey post hoc test. Number of animals in indicated in parenthesis. Representative blots are shown.

Alterations in cardiac mechanical properties and intracellular calcium handling are dependent on SERCA2a and PLB. In this study, we examined the role of these proteins in the altered myocardial contractility seen in rats 60 days after ovariectomy. As shown in Figure 3, SERCA2a protein expression is approximately 50% lower in the OVX group (Figure 3A), and PLB protein expression is increased (Figure 3B). Carvedilol treatment effectively prevented the change in SERCA2a levels but did not prevent the increase in PLB (Figure 3A and 3B). Figure 3 also demonstrates that the PLB levels in the OVX group were 1.7-fold higher than the SHAM group. The ratio of SERCA2a to PLB protein levels was significantly reduced (approximately 72%) in ovarian sex hormone-deficient hearts (Figure 3C) and improved by carvedilol treatment (Figure 3C). To examine the modulating effect of PLB on the responsiveness of SERCA2a to Ca^2+^ in ovariectomized hearts, the levels of PLB phosphorylated at either the Ser^16^ or Thr^17^ was analyzed. We did not find any difference among the groups in the content of the phosphorylated form of PLB at Ser^16^ (Figure 3E), but we did find an increase in the content of the phosphorylated form at Thr^17^ in the OVX group compared to SHAM group. Carvedilol treatment restored these changes (Figure 3F).

Furthermore, we analyzed the expression of the AT_1_ and AT_2_ receptors and we found an increase in AT_1_ receptor protein expression in the carvedilol-treated group although no was found among the other groups (Figure 3D). AT_2_ protein expression did not differ among the groups (data not shown).

### Immunohistochemical Analysis

**Figure 4:**
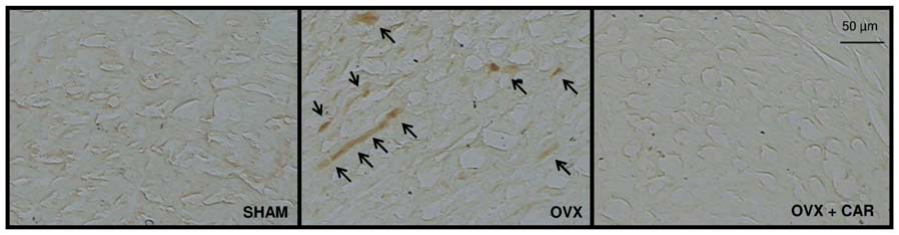
Immunohistochemistry of p22^phox^ in papillary muscle. Photomicrographs of short-axis cross-sections of papillary muscle from representative animals in the SHAM, OVX, OVX+CAR groups that were immunostained for p22^phox^ with DAB (brown). Regions of interest are shown at higher magnification (200X). Strong p22^phox^ immunoreactivity was noted in the OVX group. In carvedilol rats, p22^phox^ immunoreactivity was dramatically decreased in the papillary muscle.

Immunohistochemistry for p22^phox^ was performed in serial sections to define the spatial distribution of p22^phox^ protein expression in papillary muscle. As illustrated in Figure 4, immunoreactivity for p22^phox^ was readily detected in OVX hearts. Carvedilol treatment diminished ovariectomy-induced increase in protein expression in papillary muscle.

### Hemodynamic, Pressure-Volume Data and Collagen Content

Passive pressure-volume curve was analyzed in 2 segments: the first linear segment representing the dilatation constant, and an exponential segment indicating left ventricular stiffness. Ventricular volume or ventricular stiffness measured in the potassium-arrested heart did not differ among groups. Left ventricular dilation or ventricular stiffness was similar among all groups, showing that carvedilol treatment did not change these parameters (data not shown). We also did not find any change in the arterial or ventricular pressure (Table 2).

**Table 2:** Changes in systolic (SBP) and diastolic blood pressure (DBP), heart rate (HR), left (LVSP) ventricle systolic pressure, left ventricle diastolic pressure (LVEDP), and the positive (dP/dt max) and negative first time derivatives (dP/dt min) from SHAM, Ovariectomized (OVX) and Ovariectomized plus Carvedilol (OVX+CAR).

	SHAM	OVX	OVX+CAR
SBP (mmHg)	109±3.8	106±3.9	113±5.6
DBP (mmHg)	81±3.1	80±3	84±4.2
HR (bpm)	193±6	186±5	188±10
LVSP (mmHg)	109±3	109±3.9	121±5.1
LVEDP(mmHg)	7.6±0.7	7.8±0.7	8.3±1
dP/dtmaxLV(mmHg/sec)	4354±118	4334±145	4508±123
dP/dtmin LV(mmHg/sec)	3430±145	3388±204	3474±205

Data are shown as mean ± SEM. N = 6–9.

**Figure 5:**
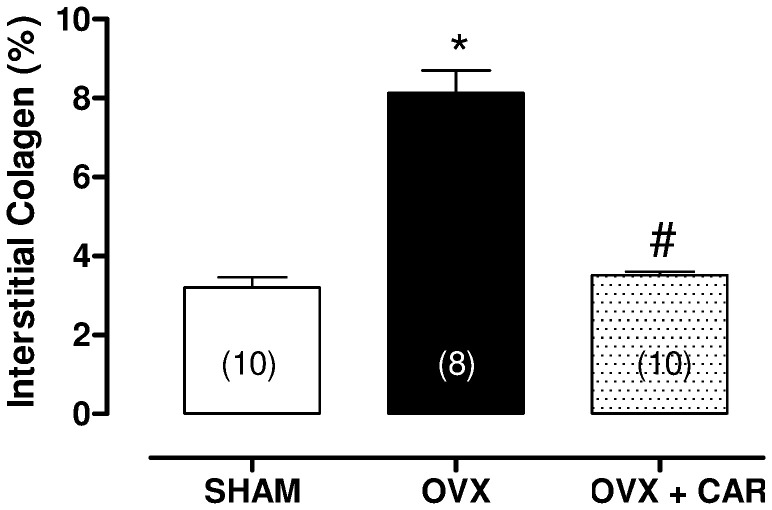
Left ventricular collagen content. The data is expressed as the mean ± S.E.M. * *p*<0.05 vs. SHAM rats. # *p*<0.05 vs. OVX rats using one-way ANOVA and Tukey post hoc test. Number of animals in indicated in parenthesis.

However, fibrosis in the spare myocardium, measured as the percentage of the area occupied by interstitial collagen, was significantly higher in the OVX group when compared to SHAM animals. Moreover, carvedilol treatment restored this increase observed in the OVX group (Figure 5).

## Discussion

Our study demonstrates that carvedilol prevented cardiac contractile dysfunction and oxidative stress after ovariectomy. This response was associated with changes in expression levels of one key protein involved in calcium handling, SERCA2a. Carvedilol normalized ovariectomy-induced contractile dysfunction, restored the SERCA2a levels and diminished reactive oxygen species formation. Furthermore, carvedilol attenuated the increase in p22^phox^ expression and affected the increase in ACE activity in the OVX group.

Clinical trials have shown that carvedilol improves heart function, morbidity, mortality rates of chronic heart failure patients and it has also been proven to be more effective than other beta blocker such as metoprolol [36]–[39]. Carvedilol has high antioxidant activity compared to any other beta blocker [22]. *In vitro* and *in vivo* studies showed that carvedilol prevents lipid peroxidation in myocardial cell membranes initiated by ROS [39]. Our results also show that carvedilol decreased lipid peroxidation even though ovariectomy did not increase plasmatic MDA levels.

Estrogen down-regulates most of the components of the renin angiotensin system, including ACE and AT_1_ receptor [4], [19], [40], [41]; therefore, estrogen deficiency leads to upregulation of AT_1_ and its affinity for angiotensin II [42]. Overexpression of angiotensin II in the heart induces cardiomyocyte remodeling such as hypertrophy, increases collagen synthesis and decreases SERCA2a expression, leading to contractile dysfunction [43], [44].

Angiotensin II most likely affects negatively excitation-contraction coupling in the myocardium by increasing superoxide production [45]. Prolonged exposure to angiotensin II activates the Ca^2+^/calmodulin–dependent protein kinase (CaMK) II and PLBThr^17^ by increasing reactive oxygen species, which leads to cell death [46].

The main source of reactive oxygen species activated by angiotensin II is the enzyme NADPH oxidase, which is associated with a smaller protein called p22^phox^ [15]. Female animals have reduced levels of p22^phox^ and superoxide anion production as well [47].This enzyme is related to cardiac remodeling and vascular dysfunction in myocardial infarction, hypertension and aging [15], [47]–[51]. Our results showed that carvedilol decreased the expression of p22^phox^, the NADPH oxidase subunit as well as superoxide anion production induced by ovariectomy. Carvedilol treatment also decreased ACE activity and increased AT_1_ receptor protein expression, in agreement with other studies, our results suggest that carvedilol down-regulates the renin angiotensin system [26], [52]–[54].

Reactive oxygen species are capable of influencing calcium homeostasis and cellular signaling via posttranslational modifications, such as S-glutathionylation, which inhibits calcium-induced calcium release in rat cardiomyocytes [55]. Reactive oxygen species can modulate calcium handling proteins [56], such as SERCA2a and L-type calcium channels, thereby contributing to slowed calcium transients [57]–[61].

Koitabashi et al [23] showed that carvedilol blocks oxidative stress-mediated downregulation of SERCA2a and suggests that carvedilol upregulates SERCA2a gene transcription by its antioxidant property. Oxidative stress may have contributed to the myocardial contractile dysfunction observed in OVX rats.

Estrogen deficiency has been related to myocardial contractile dysfunction. In agreement with other studies, our results suggest that estrogen deprivation-induced myocardial contractile dysfunction is linked to alterations in SERCA2a and PLB expression [1], [9], [10]. Phospholamban regulates SERCA2a activity by modulating calcium uptake into the sarcoplasmic reticulum and muscle relaxation [9], [27]–[29]. Based on transgenic and gene-targeted mouse model studies, alterations to the PLB to SERCA2a ratio has been suggested to be a major regulator of cardiac contractility [62]–[64].

*In vivo* studies using transgenic mice that overexpress cardiac specific PLB suggested that the “functional stoichiometry” of PLB/SERCA2 is less than 1∶1 in native cardiac sarcoplasmic reticulum membranes [62]. Here, we show that this ratio is three-fold higher in the OVX group compared to the sham group. Phospholamban is a repressor of left ventricular basal contractile parameters, and alterations in the levels of phospholamban would be expected to result in alterations in myocardial contractility. Two-fold higher PLB protein levels in transgenic mice compared to wild type resulted in decreased Ca^2+^-ATPase affinity for Ca^2+^, which was associated with decreased contractility and Ca^2+^ transport in cardiomyocytes [62]. Carvedilol treatment did not change phospholamban levels, however, it increased SERCA2a levels, thereby improving PLB/SERCA ratio.

In summary, our data indicate that treatment with carvedilol prevented the weight gain, myocardial contractile dysfunction and slight hypertrophy induced by ovariectomy. Moreover, this myocardial contractile dysfunction appeared to be dependent on superoxide anion production, which has detrimental effects on SERCA2a levels. Taken together, our findings provide evidence that carvedilol prevented myocardial contractile dysfunction in ovariectomized female rats by decreasing the renin angiotensin system activity.
